# Complete chloroplast genome sequence of *Karelinia caspia* (Pall.) Less. (Compositae)

**DOI:** 10.1080/23802359.2024.2444596

**Published:** 2024-12-25

**Authors:** Wenjuan Huang, Shuangfei Song, Chengzhi Peng, Hongyan Jin, Peipei Jiao, Zhihua Wu

**Affiliations:** a Xinjiang Production & Construction Corps Key Laboratory of Protection and Utilization of Biological Resources in Tarim Basin, College of Life Science, Tarim University, Alar, PR China; b College of Life Sciences, Zhejiang Normal University, Jinhua, PR China

**Keywords:** *Karelinia caspia* (Pall.) Less., Compositae, chloroplast genome, phylogeny

## Abstract

*Karelinia caspia* (Compositae) is a perennial herbaceous plant owning high economic, feeding and medicinal values. It is widely distributed in desertification and saline alkali areas. The complete chloroplast genome was firstly reported in this study. The chloroplast genome of K. caspia with a total size of 151,239 bp consists of two inverted repeats separated by a large single-copy region and a small single-copy region. Its chloroplast genome contains 129 genes, including 85 protein-coding genes, 36 tRNA genes, and 8 rRNA genes. Also, a total of 62 simple sequence repeats were identified. These results will be useful for study on the evolution and genetic diversity of K. caspia in the future.

## Introduction

*Karelinia caspia* (Pall.) Less. (1834) is a perennial herbaceous plant belonging to the family of Compositae, and it is a common species that widely distributed in the Gobi Desert shallows, sand dunes, saline and alkaline lands of meadows, or beside the paddy fields of Mongolia, Kazakhstan, Uzbekistan, Kyrgyzstan, Tajikistan, Turkmenistan, eastern Europe, Iran, Turkey and northwest China (Flora of China Editorial Committee, Chinese Academy of Sciences 1993). As the single species in genus *Karelinia,* it has unique properties in tolerance of drought, salinity and high temperature conditions, and thus plays essential roles in saline-alkali land improvement, windbreak, water and soil conservation and sand fixation (Wang et al. 2017). It also has certain forage value. In early spring, the tender branches and leaves are usually feed by cattle, sheep, and donkeys, and their feeding performance will be greatly improved when soaked and boiled in water, or made into hay (Luo et al. 1988). In addition, *K. caspia* is herbal medicine recorded in the list of medicinal plants in Xinjiang, involving numerous flavonoids as the major active ingredients, which have many pharmacological effects including anti-inflammation, anti-oxidation, bactericidal, anti-viral, and lowering blood lipid and blood pressure (Yang et al. 2019).

At present, the researches on *K. caspia* mainly focus on the tolerance mechanism of drought stress, salt stress and high temperature, some related genes have been cloned and their expression levels have been analyzed, such as salt-related Na^+^/H^+^ antiporter *KcNHX, KcSOS* (Zheng 2007; Pan 2008; Guo 2022), wax synthesis-related genes *KcFAD2*, *KcP450-77A* and *KcHHT* (Xu 2021; Qu et al. 2022), and thermogenesis-related gene *KcPIF4* (Huang et al. 2024). Besides, some genes have broad-spectrum resistance to either drought, salt, or high temperature, such as the vacuolar H^+^-PPase gene *KcVP1*, and *14-3-3* gene *KcHTRs* (Li 2023; Xu 2023). However, as a monospecific genus of the largest family Compositae, the chloroplast genome of *K. caspia* has not yet been reported. Due to the characteristics of uniparental inheritance, highly conserved gene content and quadripartite organization, the chloroplast genome is widely used in research on plant phylogeny and evolution studies (Wu et al. 2014). In this study, we sequenced, assembled, and annotated the accurate chloroplast genome of *K. caspia*, to obtain new insight into its phylogeny.

## Materials and methods

**Figure 1.**
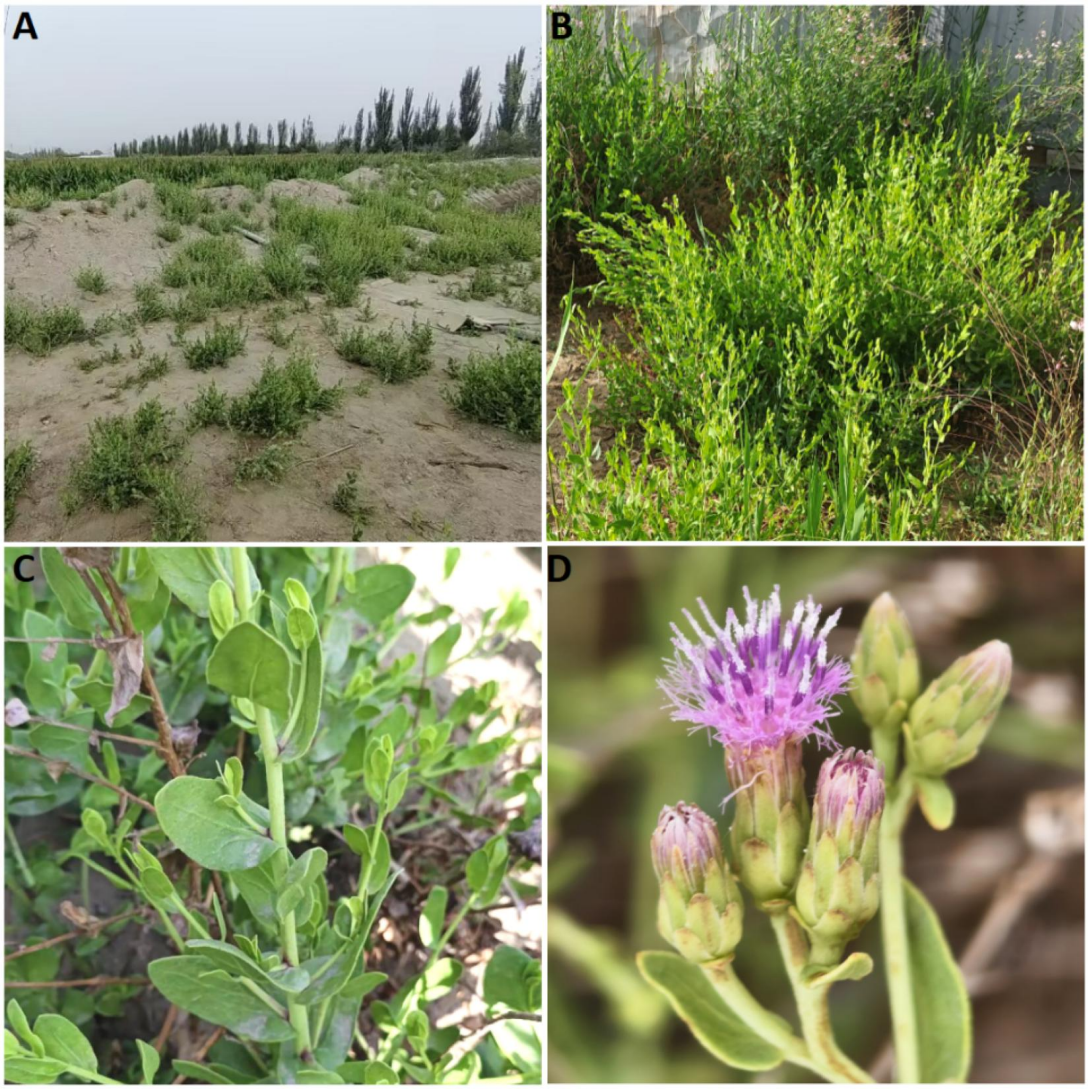
*Karelinia caspia* from Kuche County, Aksu Prefecture, Xinjiang Uygur Autonomous Region of China. (A) Growing environment (B) Whole plant morphology. (C) The branches and leaves. Multi-branched flowering stems. Succulent leaves are simple and alternate, with the basal portion of a blade embracing the stem. (D) The flower, is purple-red, multiple terminal head inflorescences arranged in cymes. The photos were taken by author Wenjuan Huang in Kuche County, Aksu Prefecture, Xinjiang Uygur Autonomous Region of China (coordinates: 83°18′18.1″E, 41°41′00.0″N).

The materials of *K. caspia* in this study were collected from Kuche County, Aksu Prefecture, Xinjiang Uygur Autonomous Region of China (83°18′18.1″E, 41°41′00.0″N, 991 m above sea level) (Figure 1). A voucher specimen (LiZJ0772, *Karelinia caspia* (Pall.) Less.), was deposited at the herbarium of Tarim University (internal website, not yet open to the public, Wenjuan Huang, hwjzky@163.com), and the data related were stored in The Germplasm Bank of Wild Species (http://www.genobank.org), data available from the corresponding author upon reasonable request. The complete genomic DNA of leaves was extracted using CTAB method (Doyle and Doyle 1987) and sequenced using the DNBSEQ-T7 platform from Huadazhizao TechnologyCo., Ltd. (Shenzhen, China). The assembly of the chloroplast genome was described as follows in brief. First, the clean reads were quality-controlled by FastQC version 0.11.9 (http://www.bioinformatics.babraham.ac.uk/projects/fastqc/). Then, the whole chloroplast genome was assembled using GetOrganelle version 1.7.3 (Jin et al. 2020). Finally, to check the accuracy of final assembly, the slimmed assembly graph and selected target assembly graph were further visualized by Bandage version 0.8.1 (Wick et al. 2015). Gene annotation was performed using CPGAVAS2 (http://47.96.249.172:16019/analyzer/annotate) (Shi et al. 2019), and PGA (https://github.com/quxiaojian/PGA) (Qu et al. 2019). Annotations of protein-coding sequences were confirmed using BLASTx in NCBI. To confirm the accuracy of the assembly, the clean reads were mapped to the assembled chloroplast genome to evaluate the coverage depth using BWA (Li and Durbin 2009). Additionally, the arrangements of genes containing introns were examined using the CPGView web platform (http://www.1kmpg.cn/cpgview, Liu et al. 2023).

To further explore the phylogenetic relationship of *K.caspia* within Compositae, additional nine species from Compositae were studied, with the *Diplostephium callilepis* S.F. Blake (1928) as the outgroup, the phylogenetic trees were built from the 57 protein-coding gene by maximum-likelihood (ML) according to the previous research (Su et al. 2023). The multiple sequence alignments were performed with MUSCLE (Multiple Sequence Comparison by Log-Expectation) version 3.8.31 (Edgar 2004), and the ML tree was generated based on concatenated alignment of single-copy genes using RAxML version 8.2.12 with DUMMY2 model and 1000 bootstrap replicates (Stamatakis 2014).

## Results

**Figure 2.**
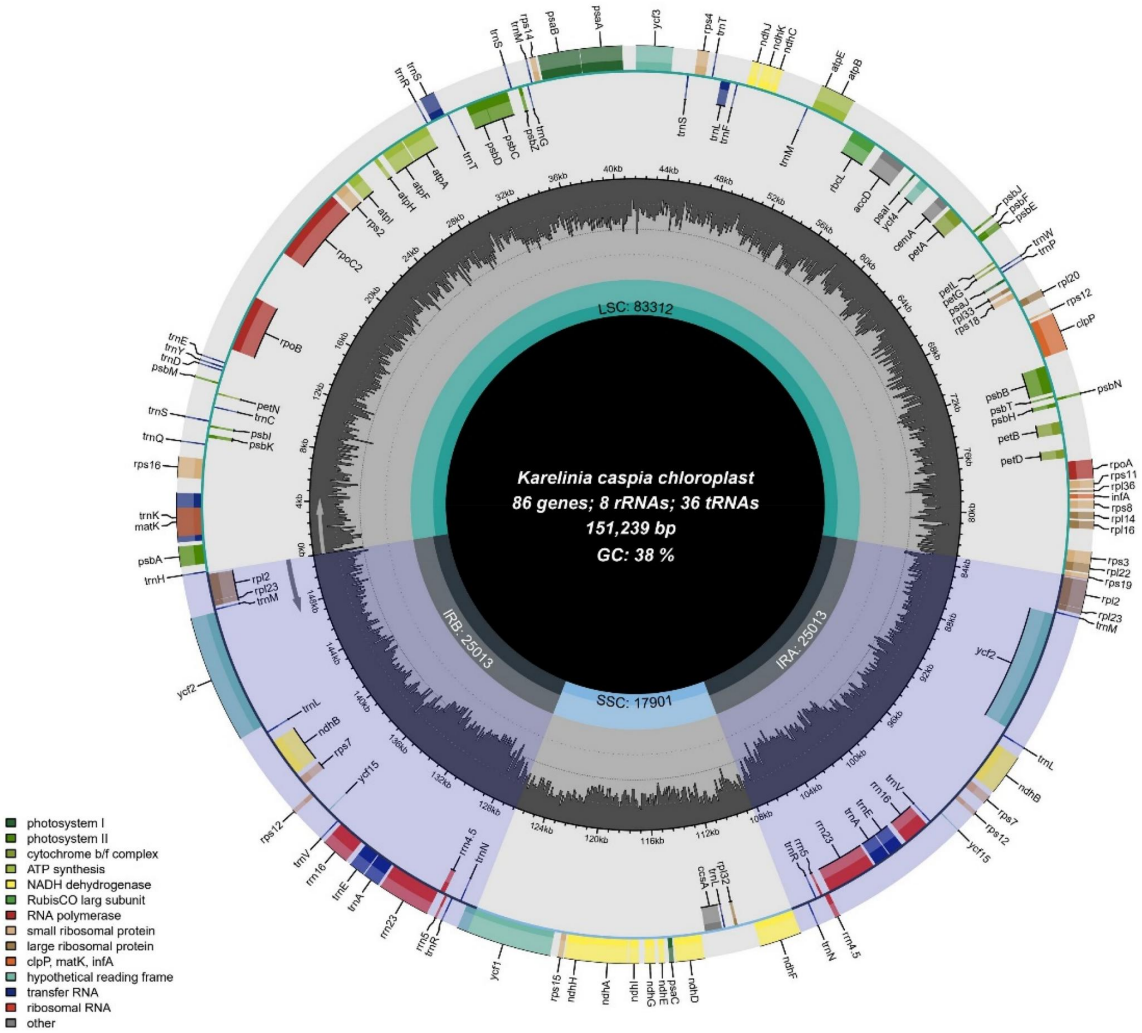
Chloroplast genome of *Karelinia caspia*. Genes inside the circle are transcribed clockwise; genes outside are transcribed counterclockwise. The dark grey inner circle corresponds to the GC content. The chloroplast genome of *Karelinia caspia* is 151,239 bp. The *Karelinia caspia* chloroplast genome contains an LSC region, an SSC region and two IR region. It encodes 129 genes, including 85 protein-coding, 8 rRNA, and 36 tRNA genes. The total GC content of the chloroplast genome was 38%.

The complete chloroplast genome was 151,239 bp and an overall GC content of 38%. The average read coverage depth for the assembled cp genome was × 498.35 (Supplementary Figure S1). The chloroplast genome of *Karelinia caspia* is composed of two IRs of 25,013 bp each, which divide into a large single copy (LSC) region of 83,312 bp and a small single copy (SSC) region of 17,901bp. The chloroplast genomes encoded 129 functional genes, including 85 protein-coding genes, 36 tRNA genes, and 8 rRNA genes (Figure 2). A total of nine cis-splicing genes were detected among the coding sequences (CDS) (Supplementary Figure S2A). Of the cis-splicing genes, seven were found to contain one intron, including *rps16, atpF, ycf3, clpP, rpl2, ndhB, ndhA,* while, two genes, *ycf3* and *clpP*, were observed to contain two introns. Additionally, the gene structure of the trans-splicing gene *rps12* was also identified (Supplementary Figure S2B). A total of 62 SSR markers including 57 mononucleotide repeat motif, two dinucleotide repeat motif, and three compound SSRs were identified in *K. caspia* chloroplast genome. The assembled and annotated cp genome has been submitted to GenBank by Chengzhi Peng (Accession no.: PQ047112).

**Figure 3.**
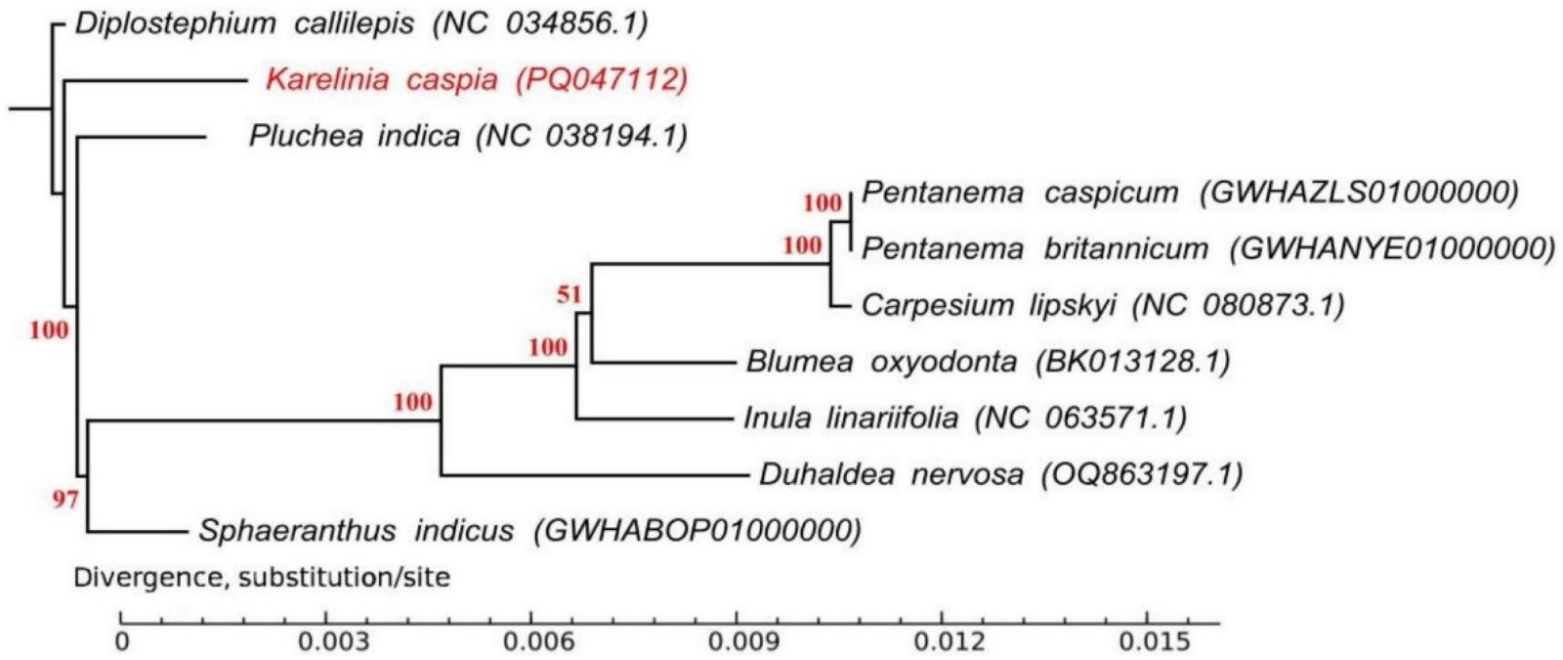
The phylogenetic tree of *Karelinia* and nine other related species was constructed based on the complete chloroplast genome sequence with maximum likelihood, and the accession numbers are shown in the figure. GenBank accession numbers are as follows: *Blumea oxyodonta BK013128.1* (Abdullah et al. 2021), *carpesium lipskyi* NC_080873.1 (Qu and Fu, 2023 Submitted), *Diplostephium callilepis* NC_034856.1 (Vargas et al. 2017), *Duhaldea nervosa* OQ863197.1 (Xing, 2023 Submitted), *Inula linariifolia* NC_063571.1 (Wu, 2022 Submitted), *Karelinia caspia* PQ047112 (this study), *Pentanema britannicum* GWHANYE01000000, *Pentanema caspicum* GWHAZLS01000000, *Pluchea indica* NC_038194.1 (Zhang et al. 2017), *Sphaeranthus indicus* GWHABOP01000000. The sequence type was chloroplast complete genome, and the accession numbers were obtained from National Center for Biotechnology Information (NCBI, https://www.ncbi.nlm.nih.gov/) or Chloroplast Genome Information Resource (CRIR, https://ngdc.cncb.ac.cn/cgir/). The accession numbers mentioned in this paper with format GWHXXXX00000000 are publicly accessible at https://ngdc.cncb.ac.cn/gwh, and the whole genome sequence data have been deposited in the Genome Warehouse (Chen et al. 2021) in National Genomics Data Center, Beijing Institute of Genomics, Chinese Academy of Sciences/China National Center for Bioinformation (CNCB-NGDC Members and Partners 2024). The bootstrap scores are reported above branches in the diagram.

A total of ten Compositae plants, including nine species from Supertrib Ambrosiodea and one species from Astereae as outgroup, were selected to construct a phylogenetic tree. The ML tree showed that all nine species from Supertrib Ambrosiodea formed one clade besides outgroup *Diplostephium callilepis*. Six species from Subtribe Inulinae (1827), and also *Pluchea indica* (1831), *Sphaeranthus indicus* (1753) from Subtribe Plucheinae grouped together, and then formed a sister group to the analyzed *K. caspia* (Figure 3).

## Discussion and conclusions

The world’s largest flowering plant family Compositae, also known as Asteraceae, comprises approximately 32,205 species that belong to 1911 genera and 13 subfamilies (http://www.theplantlist.org/1.1/browse/A/Compositae/). As a monotypic genus of subtribe Plucheinae, *K. caspia* has unique morphological characteristics and taxonomic status, the chloroplast genome information helps understanding its genome evolution. In this study, the chloroplast genome of *K. caspia* was conducted by the DNBSEQ-T7 High-throughput sequencing platform. A total of 129 genes were annotated in *K. caspia* chloroplast genome, and the genome exhibited typical quadripartite structures, including one SSC, one LSC, and a pair of IRs, like most other angiosperms. The GC content of the whole genome was 38%, which was similar to those of other species in Compositae (Vargas et al. 2017; Zhang et al. 2017;; Abdullah et al. 2021; Liang et al. 2021). The conserved genome size, structure, GC content, and organization of chloroplast genome in Compositae plants indicated the importance of chloroplasts in photosynthesis over the course of long-term plant evolution (Wicke et al. 2011; Li et al. 2017). The different gene structures may suggest that these chloroplast derived genes have diverse regulatory mechanisms (Li et al. 2020). We selected ten Compositae plants, including nine species from Supertrib Ambrosiodea and one species from Astereae as outgroup, to construct a phylogenetic tree. The ML tree showed that all nine species from Supertrib Ambrosiodea formed one clade besides outgroup *Diplostephium callilepis*. 6 species from Subtribe Inulinae, and also *Pluchea indica*, *Sphaeranthus indicus* from Subtribe Plucheinae grouped together, and then formed a sister group to the analyzed *K. caspia.* As a monotypic genus, *Karelinia* has been placed in an unresolved and morphologically complex polytomy together with a large clade includes a number of *Pluchea* species (Nylinder and Anderberg 2015), in our study, *K. caspia* also formed a sister group with *Pluchea indica*, *Sphaeranthus indicus*, and other *Inulinae* species, which was consistent with the previous study. Furthermore, *K. caspia, Pluchea indica* and *Sphaeranthus indicus* are in the same Subtribe Plucheinae based on both the classification of traditional morphology (Flora of China Editorial Committee, Chinese Academy of Sciences 1993) and APG IV system (2016) (https://duocet.ibiodiversity.net/index.php), which imply the high morphological and molecular resemblance, our results agreed well with this classification. In conclusion, our study not only enriches the genomic information of *K. caspia*, but also lays the foundation for understanding the genetic diversity, evolution, and phylogeny within the family Compositae.

## Supplementary Material

Supplementary Figures.pdf

## Data Availability

The genome sequence data that support the findings of this study are openly available in GenBank of NCBI at (https://www.ncbi.nlm.nih.gov/) under the accession no. PQ047112. The associated BioProject, SRA, and Bio-Sample numbers in National Genomics Data Center (https://ngdc.cncb.ac.cn/gsa/) are PRJCA029434, CRA018594, and SAMC4095390 respectively.”
