# Supplementary material for: Complete chloroplast genome sequence of Karelinia caspia (Pall.) Less. (Compositae)
Source: Mitochondrial DNA B Resour. 2024 Dec 25;10(1):52–6. doi: 10.1080/23802359.2024.2444596 (PMC11703384; doi:10.1080/23802359.2024.2444596)
Supplement: Supplementary Figures.pdf [file TMDN_A_2444596_SM2825.pdf]

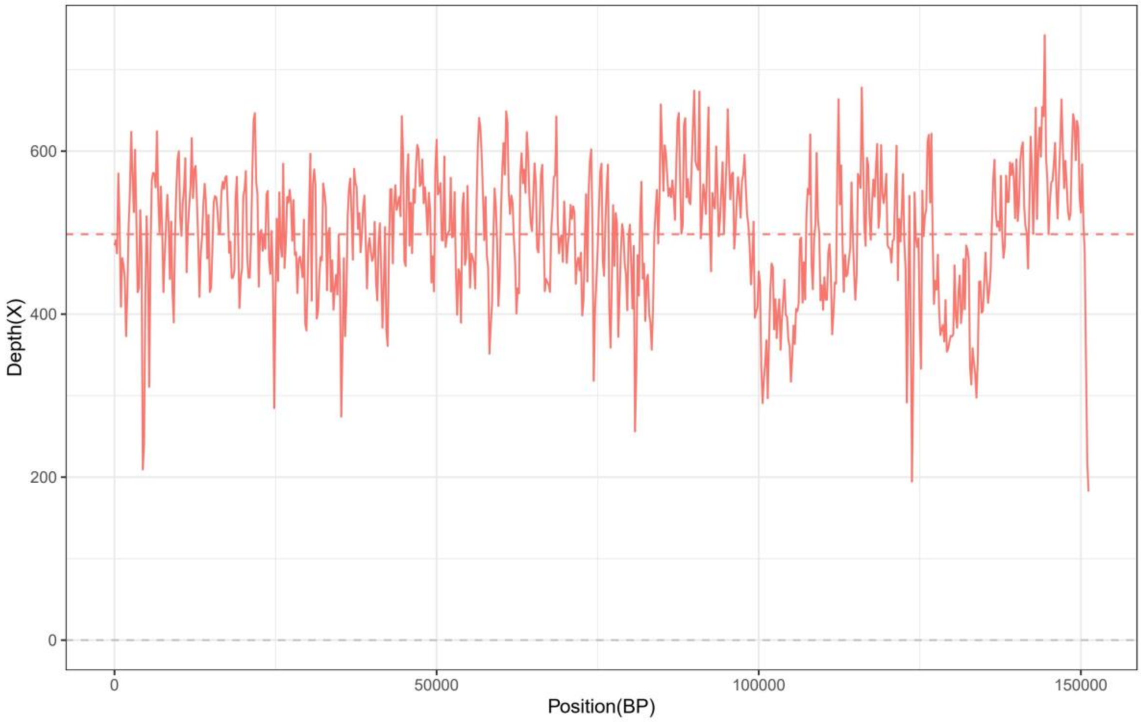

Supplementary Figure S1. The read mapping depths across the assembled chloroplast genome of *Karelinia caspia* (PQ047112).

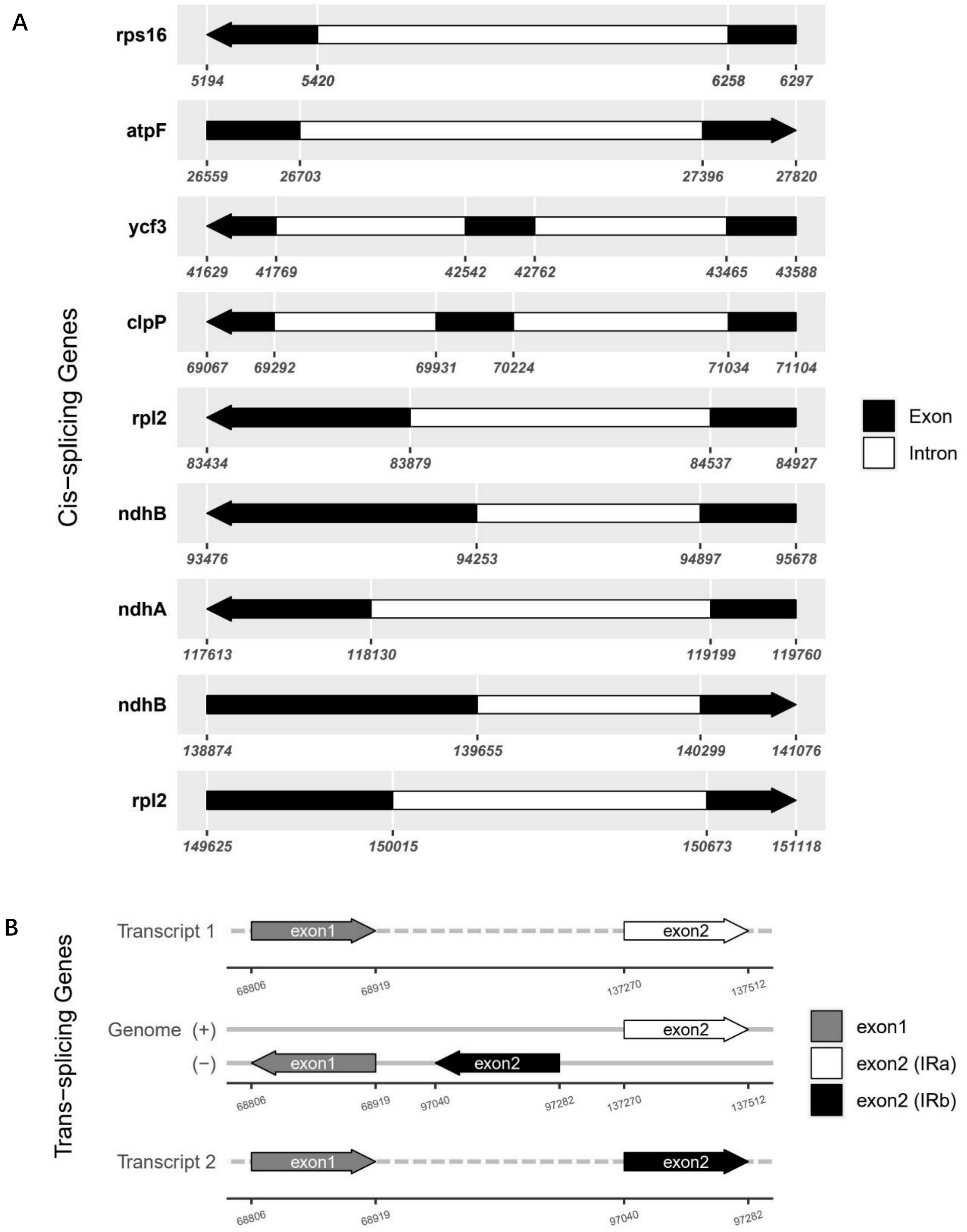

Supplementary Figure S2. Schematic map of the cis (A) and trans (B) splicing genes in the chloroplast genome of *Karelinia caspia* (PQ047112).
